# Association between Self-Reported Global Sleep Status and Prevalence of Hypertension in Chinese Adults: Data from the Kailuan Community

**DOI:** 10.3390/ijerph120100488

**Published:** 2015-01-07

**Authors:** Kai Lu, Rongjing Ding, Qin Tang, Jia Chen, Li Wang, Changying Wang, Shouling Wu, Dayi Hu

**Affiliations:** 1 Department of Cardiology, The First Affiliated Hospital of Chongqing Medical University, Chongqing 400016, China; E-Mails: lukai2013@foxmail.com (K.L.); drchenjia@foxmail.com (J.C.); wangli514@sina.cn (L.W.); wangchangying04@163.com (C.W.); dayihu2014@163.com (D.H.); 2 Heart Center, Peking University People’s Hospital, No.11 South Xizhimen Avenue, Beijing 100044, China; 3 Department of Education and Science, China Medical Association, Beijing 100044, China; E-Mail: drtangqin@163.com; 4 Department of Cardiology, The Kailuan General Hospital, Hebei United University, No.57, East Xinhua Avenue, Tangshan 063001, China

**Keywords:** hypertension, sleep status, sleep quality, sleep duration

## Abstract

*Background:* Assessment of sleep only by sleep duration is not sufficient. This cross-sectional study aimed to investigate the potential association of self-reported global sleep status, which contained both qualitative and quantitative aspects, with hypertension prevalence in Chinese adults. *Methods:* A total of 5461 subjects (4076 of them were male) were enrolled in the current study and were divided into two groups with the age of 45 years as the cut-off value. Sleep status of all subjects was assessed using the standard Pittsburgh Sleep Quality Index (PSQI). Hypertension was defined as blood pressure ≥140/90 mmHg in the current study. *Results:* After adjusting for basic cardiovascular characteristics, the results of multivariate logistic regression indicated that sleep status, which was defined as the additive measurement of sleep duration and sleep quality, was associated with hypertension prevalence in males of both age groups (odds ratio (OR) = 1.11, 95% confidence interval (CI), 1.07–1.15, *p* < 0.05; OR = 1.12, 95% CI, 1.08–1.15, *p* < 0.05) and in females aged ≤45years (OR = 1.10, 95% CI, 1.02–1.18, *p* < 0.05). As one component of PSQI, short sleep duration was associated with hypertension prevalence only in Chinese male subjects, but this association disappeared after the further adjustment of the other components of PSQI that measured the qualitative aspect of sleep. *Conclusion:* Association between sleep status and hypertension prevalence in Chinese adults varied by age and sex. Sleep should be measured qualitatively and quantitatively when investigating its association with hypertension.

## 1. Introduction

The relationship between hypertension and short sleep duration has raised concerns of cardiologists in recent years. Dozens of cross-sectional [1,2] and longitudinal studies [3,4,5] have been conducted in populations of different ages, genders or races to investigate the potential association of short sleep duration in relation to hypertension and most of them indicated short sleep duration was related with the prevalence and incidence of hypertension. However, a few studies noted that sleep had quantitative and qualitative aspects [6,7] and it may be not comprehensive to assess sleep only by sleep duration in those studies. Particularly, Bansil* et al.*’s study, which investigated the role of sleep duration and sleep quality in hypertension development separately, found that sleep duration alone failed to affect the hypertension prevalence, whereas the combination of short sheep duration and sleep disorders could influence hypertension [7]. Furthermore, the literature documented that poor sleep quality was associated with obesity [8,9], metabolic syndrome [10] and glucose metabolism [11] which share many common risk factors with hypertension. Those findings suggested a possible link between sleep quality and hypertension which, however, has not been fully elucidated in the current literature. Therefore, it is necessary to investigate the potential relationship between prevalence of hypertension and global sleep status which contains both quantitative and qualitative aspects.

Pittsburgh Sleep Quality Index (PSQI) is a widely used tool for measurement of global sleep status and has acceptable internal homogeneity, test-retest reliability, and validity for clinical practice and research [12]. In the current study, we investigated the potential association between prevalence of hypertension and global sleep status assessed by PSQI using the data of a cross-sectional survey in Chinese adults.

## 2. Study Design and Population

This cross-sectional study was conducted from September to December 2013 in the Kailuan community which is located in Tangshan, a northern city of China. The Kailuan community is a functional and comprehensive community which contains ten sub-communities owned and managed by the Kailuan Group. More information about the Kailuan community can be found elsewhere [13,14]. In the present study, four sub-communities, Tangshan, Fangezhuang, Lvjiatuo and Qianjiaying, were selected randomly from the Kailuan community. Subjects aged 18 years or more in those four sub-communities were invited to participate into this study. Critical exclusion criteria included: diagnosed or suspected secondary hypertension; hypertension in pregnancy; severe chronic heart failure; severe liver dysfunction; end-stage renal disease; advanced cancer; and previous diagnosis of obstructive sleep apnea syndrome (OSAS) or restless legs syndrome (RLS). In addition, subjects with a score of 1 or more on the item 10 of the PSQI, which indicated a high likelihood of comorbidity of OSAS, were excluded from the final analysis after enrollment. The contents and purposes of this study were thoroughly explained to the participants prior to the initiation of the study, and written consents were obtained from all participants.

The study protocols were in accordance with the Declaration of Helsinki and the ethical approval was obtained from the Science and Technology Committee of Tangshan City.

## 3. Methods and Measurements

### 3.1. Anthropometric Measurements

Height and weight was measured to the nearest 0.1 cm or 0.1 kg when the subjects stood upright and barefoot in light clothes. Two separate measurements of height and weight for each subject were performed and the average was used for analysis. Body mass index (BMI) was calculated as the ratio of weight to height squared (kg/m^2^). Blood pressure was measured twice using standard mercury sphygmomanometers (Yuyue, China) in a seated position with a 5 min interval after a resting period of 10 min. Average of the two measurements was recorded as the final blood pressure. However, when the systolic or diastolic pressures exhibited a difference greater than 5 mmHg, a third measurement was necessary and the final blood pressure value was recorded as the average of the three measurements. Hypertension was defined according to the 7th edition report of the USA Joint National Committee on Prevention, Detection, Evaluation and Treatment of Hypertension [15]: as SBP ≥ 140 mmHg and/or DBP ≥ 90 mmHg on average of measurements or by current antihypertensive treatment.

### 3.2. Blood Test

Subjects were asked to fast overnight before venous blood sample collection. Blood was collected from antecubital veins and then centrifuged at 3000 rpm for 10 min to isolate plasma. The supernatant serum were tested within 4 h in the central laboratory of Kailuan Hospital on automatic biochemical analyzers (Hitachi 717, Tyoko, Japan) for concentrations of total triglyceride (TG), total cholesterol (TC) and fasting blood glucose (FBG). Kit was provided by the Biology Institute of North China.

### 3.3. Questionnaire Survey

A questionnaire survey was conducted face to face on paper to obtain demographic and behavior-associated information such as age, gender, status of smoking, status of drinking, and physical exercise habits. Status of smoking and drinking was classified as “never”, “former”, or “current” using the self-reported information. Exercise activity was evaluated from responses to questions about the type and frequency of physical activity during leisure time and was classified as “active” and “inactive”. Subjects with at least 30 min physical activities for at least 5 days per week were defined as active exercisers. Sleep status of the participants was assessed by the Chinese version of the Pittsburgh Sleep Quality Index (PSQI) scale. PSQI scale consists of seven elements (subjective sleep quality, sleep latency, sleep duration, habitual sleep efficiency, sleep disturbance, use of sleep medication, and daytime dysfunction) and each of them consisted of a four-grade system (*i.e*., 0, 1, 2, 3) [12]. The total score of PSQI is 21 and any participant with a score of 6 or more was diagnosed as having a sleep disorder. According the study of Tsai* et al.* [16], the Chinese version of the PSQI has good overall reliability (r = 0.82–0.83) and test–retest reliability (r = 0.77–0.85).

In addition, considering the frequent comorbidity of sleep disorders with anxiety and depression which also exert an influence on the prevalence of hypertension, status of anxiety and depression of participants were evaluated by the General Anxiety Disorder-7 (GAD-7) and Patient Health Questionnaire-9 (PHQ-9), respectively. GAD-7 is a seven-question inventory for self-assessment and is one of the most common instruments for measuring the severity of anxiety [17]. PHQ-9 is a widely used nine-question inventory for self-assessment of depression [18]. Both of the cut-off points to define anxiety and depression were ≥5 on GAD-7 or PHQ-9 [19,20].

## 4. Statistics

All statistical analyses were conducted separately by gender because significant interaction of gender and sleep status on hypertension prevalence was detected (F = 3.48, *p* < 0.05). Moreover, previous reports indicated that the associations of sleep in relation to hypertension varied with the ages of participants [2,3,21]; we therefore divided the participants into two groups with the age of 45 years as the cut-off value based on previous reports [2] and the age range of the present study (18 years to 72 years, with the average of 46.2). In addition, significant interaction between the two age classes for prevalence of hypertension was also observed in the present study (F = 2.19, *p* < 0.05). Continuous variables were presented as mean ± standard deviation (SD) and the comparisons between two groups were tested by *t*-tests or non-parametric tests based on distributional properties. Categorical variables were presented as frequencies and proportions and the comparisons between two groups were conducted via chi-square (χ^2^) test. To explore the potential association of sleep status as well as its seven components in relation to hypertension prevalence, multivariate logistic analysis was used and other cardiovascular characteristics including age, body mass index (BMI), triglycerides (TG), total cholesterol (TC), fasting blood glucose (FBG), physical activity, status of smoking, status of drinking, score of GAD-7 and PHQ-9 were adjusted for. Considering that the limited number of subjects with the grade of 3 on the components of PSQI was not enough for multivariate logistic regression where a couple of confounders were adjusted, subjects with grades of 2 and 3 were merged. To investigate the specific association of sleep duration with hypertension prevalence, the total scores of the other six components of PSQI were further adjusted for on the basis of the adjustment of cardiovascular characteristics. For all the comparisons, the level of statistical significance was set at *p* < 0.05. All statistical analyses were conducted by SPSS 19.0 (IBM, Armonk, NY, USA).

## 5. Results

There were 5980 citizens invited for the current study and 519 of them were precluded due to missing data. A total of 4076 males and 1385 females were finally enrolled into the current study. Male/female subjects were divided into two groups according to age ≤45 or >45 years. The basic characteristics of those subjects with or without hypertension are presented in Table 1.

### 5.1. Scores of Global PSQI and Its Components in Hypertensive and Normotensive Subjects

Scores of global PSQI and each component of it in subjects with or without hypertension are presented in Table 2. In male subjects aged ≤45 and >45 years old, hypertensive ones received significantly higher scores of global PSQI (4.30 ± 0.12* vs.*3.30 ± 0.07; 5.44 ± 0.24* vs.* 4.16 ± 0.11) and had a higher prevalence of sleep disorder (34.5%* vs.* 23.7%; 32.4%* vs.* 18.2%) than normotensive men. Out of the seven components of PSQI in males aged ≤45 years, hypertensive subjects had higher scores on six of them with the exception of sleep efficiency as compared with normotensive subjects. However, in male subjects aged >45 years, hypertensive men received higher scores on only five of the PSQI components and no significant statistical differences were found as to the scores of sleep efficiency and sleep latency between subjects with and without hypertension.

For female subjects aged ≤45 years, hypertensive women had significantly higher scores of global PSQI (5.73 ± 0.42* vs.* 4.52 ± 0.26) and higher prevalence of sleep disorders (54.3%* vs.* 29.6%) than normotensive women, which was similar to their male counterparts. In this group of participants, however, subjects with hypertension received higher scores on only one of the seven components of PSQI, the subjective sleep quality, in comparison to normotensive women. An obvious discrepancy for female subjects aged >45 years was observed between them and their male counterparts. With the exception of higher prevalence of sleep disorders in hypertensive subjects, no significant differences between hypertensive and normotensive subjects were found with regards to the scores of global PSQI and its seven components.

**Table 1:** The basic characteristics of male and female participants.

Basic Cardiovascular Characteristics	≤45 Years (N = 1934)				>45 years (N =2142)			
	Non-Hypertension	Hypertension	*p*	Total	Non-Hypertension	Hypertension	*p*	Total
**Males**								
	**N = 1433**	**N = 501**	**-**	**N = 1934**	**N = 1530**	**N = 612**	**-**	**N = 2142**
Age (year)	39.45 ± 5.15	40.62 ± 4.89	0.00	39.76 ± 5.08	50.91 ± 9.26	51.29 ± 3.98	0.27	51.43 ± 8.03
BMI (kg/m^2^)	25.32 ± 3.88	26.21 ± 3.87	0.00	25.54 ± 3.90	24.85 ± 3.36	25.77 ± 3.41	0.00	25.13 ± 3.40
SBP (mmHg)	125.41 ± 13.02	132.77 ± 13.39	0.00	127.26 ± 13.51	129.48 ± 14.61	135.91 ± 14.64	0.00	131.62 ± 14.84
DBP (mmHg)	83.33 ± 8.85	89.26 ± 9.20	0.00	84.76 ± 9.31	85.06 ± 9.41	89.78 ± 10.11	0.00	86.47 ± 9.83
TC (mmol/L)	4.82 ± 0.94	4.94 ± 0.91	0.04	4.84 ± 0.94	4.90 ± 0.93	4.15 ± 0.97	0.02	4.95 ± 0.94
TG (mmol/L)	1.95 ± 2.02	2.19 ± 2.38	0.03	2.01 ± 2.11	1.85 ± 1.70	2.11 ± 2.18	0.00	1.91 ± 1.79
FBG (mmol/L)	5.19 ± 1.05	5.38 ± 1.40	0.01	5.23 ± 1.15	5.54 ± 1.52	5.69 ± 1.75	0.03	5.58 ± 1.62
Active exercise habits (%)	444 (31.0)	117 (23.3)	0.00	561 (29.0)	568 (37.1)	174 (28.5)	0.00	742 (34.3)
Current smoker (%)	745 (52.0)	291 (58.0)	0.00	1,036 (53.5)	805 (52.6)	390 (63.8)	0.00	1,195 (55.8)
Current drinker (%)	555 (38.7)	222 (44.4)	0.00	777 (40.2)	644 (42.1)	297 (48.6)	0.00	941 (43.9)
GAD-7	2.29 ± 0.11	2.81 ± 0.24	0.03	2.41 ± 0.10	2.12 ± 0.10	2.50 ± 0.15	0.01	2.16 ± 0.09
PHQ-9	2.55 ± 0.12	3.50 ± 0.27	0.00	2.78 ± 0.11	2.10 ± 0.10	2.62 ± 0.06	0.01	2.26 ± 0.09
**Females**								
	**N = 695**	**N = 140**	**-**	**N = 835**	**N = 380**	**N = 170**	**-**	**N = 550**
Age (year)	40.29 ± 3.78	41.56 ± 3.51	0.00	40.53 ± 3.74	48.55 ± 4.21	49.35 ± 5.16	0.03	49.91 ± 4.46
BMI (kg/m^2^)	25.31 ± 4.64	25.58 ± 4.76	0.29	25.40 ± 4.63	25.09 ± 5.31	25.75 ± 3.72	0.21	25.04 ± 3.47
SBP (mmHg)	127.03 ± 17	130.85 ± 13.26	0.00	127.60 ± 16.59	123.72 ± 16.86	130.22 ± 16.97	0.00	126.21 ± 16.84
DBP (mmHg)	82.47 ± 9.24	83.45 ± 8.67	0.12	82.71 ± 9.16	80.81 ± 10.68	85.61 ± 12.45	0.00	82.46 ± 11.35
TC(mmol/L)	4.88 ± 0.90	5.00 ± 0.79	0.13	4.90 ± 0.89	5.00 ± 0.93	5.04 ± 1.01	0.52	4.98 ± 0.96
TG (mmol/L)	1.80 ± 1.44	1.72 ± 1.04	0.28	1.86 ± 1.59	1.96 ± 1.58	1.78 ± 1.30	0.26	1.88 ± 1.34
FBG (mmol/L)	5.65 ± 1.99	5.14 ± 0.91	0.02	5.56 ± 1.86	5.43 ± 0.84	5.28 ± 1.08	0.14	5.42 ± 1.11
Active exercise habits (%)	221 (31.8)	54 (38.3)	0.12	275 (32.9)	144 (37.9)	64 (37.6)	0.96	208 (37.8)
Current smoker (%)	10 (1.5)	6 (4.0)	0.00	16 (1.9)	5 (1.4)	14 (5.1)	0.00	19 (3.5)
Current drinker (%)	166 (23.9)	39 (28.0)	0.33	205 (24.6)	105 (27.6)	52 (30.7)	0.50	274 (49.8)
GAD-7	3.77 ± 0.44	4.39 ± 0.57	0.00	3.86 ± 0.41	2.38 ± 0.65	3.91 ± 0.70	0.00	3.37 ± 0.51
PHQ-9	3.91 ± 0.46	6.33 ± 0.52	0.00	4.23 ± 0.47	3.30 ± 0.69	3.74 ± 0.83	0.22	3.57 ± 0.57

BMI, body mass index; SBP, systolic blood pressure; DBP, diastolic blood pressure; TC, total cholesterol; TG, triglycerides; FBG, fasting blood glucose; GAD-7, General Anxiety Disorder-7; PHQ-9, Patient Health Questionnaire.

**Table 2:** Scores of global PSQI and its component in both male and female subjects with and without hypertension.

PSQI and Its Components	≤45 Years (N= 1934)				>45 years (N= 2142)			
	Non-Hypertension	Hypertension	*p*	Total	Non-Hypertension	Hypertension	*p*	Total
**Male**								
Global PSQI score	**3.30 ± 0.07**	**4.30 ± 0.12**	**0.00**	**3.61 ± 0.06**	**4.16 ± 0.11**	**5.44 ± 0.24**	**0.00**	**4.46 ± 0.11**
Sleep disorder (n, %) (PSQI score ≥ 6)	**340 (23.7)**	**173 (34.5)**	**0.00**	**513 (26.5)**	**278 (18.2)**	**198 (32.4)**	**0.00**	**476 (22.2)**
Subjective sleep quality	**0.53 ± 0.02**	**0.74 ± 0.03**	**0.00**	**0.60 ± 0.02**	**0.63 ± 0.02**	**0.76 ± 0.05**	**0.01**	**0.66 ± 0.02**
Sleep latency	**0.23 ± 0.01**	**0.37 ± 0.03**	**0.00**	**0.27 ± 0.01**	0.85 ± 0.04	1.09 ± 0.07	0.52	0.90 ± 0.03
Sleep duration	**0.41 ± 0.02**	**0.57 ± 0.03**	**0.00**	**0.46 ± 0.01**	**0.39 ± 0.02**	**0.53 ± 0.04**	**0.01**	**0.43 ± 0.02**
Habitual sleep efficiency	0.40 ± 0.02	0.39 ± 0.04	0.69	0.40 ± 0.02	0.36 ± 0.02	0.39 ± 0.04	0.46	0.36 ± 0.02
Sleep disturbance	**0.56 ± 0.02**	**0.79 ± 0.02**	**0.00**	**0.63 ± 0.01**	**0.59 ± 0.02**	**0.82 ± 0.04**	**0.00**	**0.65 ± 0.02**
Use of sleep medication	**0.74 ± 0.02**	**0.89 ± 0.03**	**0.00**	**0.79 ± 0.01**	**0.76 ± 0.02**	**0.92 ± 0.04**	**0.00**	**0.80 ± 0.02**
Daytime dysfunction	**0.39 ± 0.02**	**0.53 ± 0.03**	**0.00**	**0.43 ± 0.01**	**0.75 ± 0.03**	**0.98 ± 0.07**	**0.00**	**0.81 ± 0.03**
**Female**								
Global PSQI score	**4.52 ± 0.26**	**5.73 ± 0.42**	**0.01**	**4.88 ± 0.22**	4.78 ± 0.23	5.12 ± 0.37	0.44	4.87 ± 0.19
Sleep disorder (n, %) (PSQI score ≥ 6)	**206 (29.6)**	**76 (54.3)**	**0.00**	**282 (33.8)**	**115 (30.2)**	**61 (35.9)**	**0.00**	**176 (32.0)**
Subjective sleep quality	**0.92 ± 0.05**	**1.11 ± 0.08**	**0.03**	**0.98 ± 0.04**	0.93 ± 0.06	1.14 ± 0.11	0.07	0.99 ± 0.05
Sleep latency	0.71 ± 0.04	0.85 ± 0.07	0.07	0.75 ± 0.04	0.78 ± 0.05	0.93 ± 0.09	0.13	0.82 ± 0.04
Sleep duration	0.35 ± 0.04	0.38 ± 0.06	0.64	0.36 ± 0.03	0.39 ± 0.05	0.51 ± 0.09	0.19	0.42 ± 0.04
Habitual sleep efficiency	0.33 ± 0.04	0.33 ± 0.06	0.99	0.33 ± 0.03	0.51 ± 0.06	0.31 ± 0.08	0.07	0.46 ± 0.05
Sleep disturbance	0.35 ± 0.14	0.55 ± 0.20	0.43	0.41 ± 0.11	0.41 ± 0.04	0.23 ± 0.12	0.06	0.36 ± 0.04
Use of sleep medication	0.89 ± 0.04	0.95 ± 0.06	0.42	0.91 ± 0.03	0.96 ± 0.04	1.06 ± 0.08	0.25	0.98 ± 0.04
Daytime dysfunction	**0.83 ± 0.04**	**1.00 ± 0.06**	**0.02**	**0.88 ± 0.03**	0.80 ± 0.05	0.94 ± 0.08	0.16	0.84 ± 0.04

PSQI, Pittsburgh Sleep Quality Index.

### 5.2. The Association between Sleep Status and Hypertension Prevalence

The results of multivariate logistic regression analyses about the association between sleep status (global PSQI score and its components) and hypertension prevalence are presented in Table 3 (for males) and Table 4 (for females). After adjusting for age, BMI, TC, TG, FBG, physical exercise, status of smoking and drinking, scores of GAD-7 and PHQ-9, global PSQI score was found to be associated with the prevalence of hypertension in male subjects aged ≤45 and >45 years and the corresponding odds ratio (OR) was 1.11 (95% confidence interval (CI), 1.07–1.15) and 1.12 (95% CI, 1.08–1.15), respectively. Sleep disorder was also found to be in relation to hypertension prevalence in male subjects aged ≤45 (OR = 1.79, 95% CI, 1.37–2.35) and >45 (OR = 1.86, 95% CI, 1.51–2.30). In addition, our results demonstrated that prevalence of hypertension was associated with five components of PSQI in male subjects of both age groups: subjective sleep quality, sleep latency, sleep duration, sleep disturbance, use of sleep medication and daytime dysfunction.

For female subjects aged ≤45 years, global PSQI score and sleep disorder were also significantly associated with the prevalence of hypertension (OR = 1.10, 95% CI, 1.02–1.18; OR = 1.84, 95% CI, 1.06–3.19), which was similar to their male counterparts. However, global PSQI score and sleep disorder were not associated with hypertension prevalence in female subjects aged >45 years. As for the association of components of PSQI in relation to hypertension prevalence in females, none of them was found to be statistically significant. In contrast to the result from their male counterparts, short sleep duration failed to be associated with hypertension prevalence in females aged either ≤45 years and >45 years.

### 5.3. Association of Sleep Duration with Hypertension Prevalence after Adjustment of the Qualitative Aspect of Sleep

To preclude the potential interaction of sleep quality and sleep duration and investigate the specific association of sleep duration with prevalence of hypertension in male subjects, the scores of the other six components of PSQI which measured the qualitative aspect of sleep were further adjusted for addition to the basic cardiovascular characteristics in the multivariate logistic regression analysis. As shown in Table 5, sleep duration was no longer significantly associated with hypertension prevalence in both age groups. The results were inconsistent with the results demonstrated in Table 3 where qualitative components of PSQI were not adjusted for.

**Table 3:** Odds ratios (OR) of scores of PSQI and its components for prevalence of hypertension in male subjects.

PSQI and Its Components	Grade	N	≤45 years (N= 1934)		N	>45 years (N = 2142)	
			Unadjusted OR (95% CI)	Adjusted OR (95% CI) ^†^		Unadjusted OR (95%)	Adjusted OR (95% CI)^ †^
Global PSQI score		**1934**	**1.11 (1.07–1.14)**	**1.11 (1.07–1.15)**	**2142**	**1.12 (1.09–1.15)**	**1.12 (1.08–1.15)**
Sleep disorder (PSQI score ≥ 6)	No	1421	1.00 (Reference)	1.00 (Reference)	1666	1.00 (Reference)	1.00 (Reference)
	Yes	**513**	**1.72 (1.32–2.30)**	**1.79 (1.37–2.35)**	**476**	**1.95 (1.59–2.39)**	**1.86 (1.51–2.30)**
Subjective sleep quality	0	1031	1.00 (Reference)	1.00 (Reference)	1268	1.00 (Reference)	1.00 (Reference)
	1	619	1.04 (0.79–1.37)	1.07 (0.80–1.42)	553	1.25 (1.02–1.52)	1.26 (1.02–1.54)
	2–3	**284**	**1.61 (1.09–2.36)**	**1.62 (1.09–2.42)**	**321**	**1.68 (1.28–2.21)**	**1.63 (1.23–2.17)**
Sleep latency	0	940	1.00 (Reference)	1.00 (Reference)	1694	1.00 (Reference)	1.00 (Reference)
	1	777	1.11 (0.86–1.45)	1.13 (0.86–1.49)	334	1.38 (1.10–1.73)	1.35 (1.06–1.71)
	2–3	**217**	**2.15 (1.46–3.16)**	**2.28 (1.52–3.40)**	**114**	**2.15 (1.52–3.11)**	**2.18 (1.50–3.18)**
Sleep duration	0	1,298	1.00 (Reference)	1.00 (Reference)	1403	1.00 (Reference)	1.00 (Reference)
	1	487	1.13 (0.85–1.50)	1.18 (0.88–1.58)	536	1.24 (1.02–1.51)	1.22 (0.99–1.50)
	2–3	**149**	**2.50 (1.61–3.89)**	**2.62 (1.65–4.15)**	**203**	**1.96 (1.44–2.67)**	**1.90 (1.38–2.62)**
Habitual sleep efficiency	0	1509	1.00 (Reference)	1.00 (Reference)	1555	1.00 (Reference)	1.00 (Reference)
	1	239	1.25 (0.87–1.80)	1.20 (0.82–1.76)	283	1.09 (0.84–1.42)	1.12 (0.86–1.46)
	2–3	186	1.00 (0.55–1.82)	1.05 (0.57–1.92)	304	1.08 (0.76–1.54)	1.148 (0.80–1.64)
Sleep disturbance	0	837	1.00 (Reference)	1.00 (Reference)	966	1.00 (Reference)	1.00 (Reference)
	1	**969**	**1.71 (1.31–2.24)**	**1.75 (1.32–2.31)**	**1035**	**2.04 (1.70–2.44)**	**2.01 (1.67–2.42)**
	2–3	**128**	**3.30 (1.99–5.47)**	**3.53 (2.08–6.00)**	**141**	**3.14 (2.22–4.46)**	**3.29 (2.29–4.74)**
Use of sleep medication	0	698	1.00 (Reference)	1.00 (Reference)	743	1.00 (Reference)	1.00 (Reference)
	1	**977**	**1.35 (1.03–1.77)**	**1.42 (1.07–1.88)**	**1148**	**1.35 (1.11–1.63)**	**1.34 (1.10–1.63)**
	2–3	**259**	**1.66 (1.11–2.47)**	**1.82 (1.20–2.76)**	**251**	**1.98 (1.48–2.65)**	**2.13 (1.58–2.87)**
Daytime dysfunction	0	1135	1.00 (Reference)	1.00 (Reference)	1401	1.00 (Reference)	1.00 (Reference)
	1	**594**	**1.33 (1.02–1.73)**	**1.38 (1.05–1.83)**	**568**	**1.59 (1.31–1.93)**	**1.56 (1.28–1.90)**
	2–3	**205**	**1.57 (1.06–2.32)**	**1.56 (1.03–2.36)**	**173**	**1.66 (1.21–2.28)**	**1.60 (1.14–2.22)**

PSQI, Pittsburgh Sleep Quality Index. CI, confidence interval; **^†^** Adjusting for age, BMI, TC,TG, FBG, exercise habit, status of smoking, status of drinking, score of GAD-7 and PHQ-9.

**Table 4:** Odds ratios (OR) of scores of PSQI and its components for prevalence of hypertension in female subjects.

PSQI and Its Components	Grade	N	≤45 years (N = 835)		N	>45 years (N = 550)	
			UnadjustedOR (95%)	AdjustedOR (95% CI) ^†^		Unadjusted OR (95%)	Adjusted OR (95% CI) ^†^
Global PSQI score		**835**	0.98 (0.88–1.10)	1.10 (1.02–1.18)	550	0.93 (0.75–1.17)	1.03 (0.96–1.1)
Sleep disorder	No	**529**	1.00 (Reference)	1.00 (Reference)	332	1.00 (Reference)	1.00 (Reference)
(PSQI score ≥ 6)	Yes	**306**	**1.76 (1.17–2.61)**	**1.84 (1.06–3.19)**	218	1.34 (0.43–2.27)	1.36 (0.80–2.30)
Subjective sleep quality	0	256	1.00 (Reference)	1.00 (Reference)	188	1.00 (Reference)	1.00 (Reference)
	1	337	**1.22 (1.02–1.46)**	1.24 (0.78–1.95)	207	1.42 (0.74–2.59)	1.39 (0.77–2.54)
	2–3	242	1.30 (0.67–2.51)	1.32 (0.68–2.54)	155	1.47 (0.45–4.95)	1.47 (0.69–3.11)
Sleep latency	0	209	1.00 (Reference)	1.00 (Reference)	233	1.00 (Reference)	1.00 (Reference)
	1	232	3.72 (0.51–27.90)	3.72 (0.46–30.13)	191	1.40 (0.70–2.81)	1.43 (0.81–2.50)
	2–3	394	4.11 (0.49–35.87)	4.11 (0.48–34.84)	126	1.53 (0.50–4.75)	1.55 (0.67–3.56)
Sleep duration	0	625	1.00 (Reference)	1.00 (Reference)	396	1.00 (Reference)	1.00 (Reference)
	1	144	1.38 (0.52–3.67)	1.37 (0.83–2.26)	116	1.64 (1.21–2.15)	1.68 (0.94–3.03)
	2–3	66	0.95 (0.43–2.12)	0.97 (0.39–2.43)	48	0.91 (0.24–2.86)	0.95 (0.30–3.01)
Habitual sleep efficiency	0	670	1.00 (Reference)	1.00 (Reference)	397	1.00 (Reference)	1.00 (Reference)
	1	90	0.90 (0.19–4.35)	0.83 (0.44–1.60)	69	0.70 (0.31–1.55)	0.64 (0.28–1.46)
	2–3	75	1.64 (0.69–3.46)	1.66 (0.70–3.90)	84	0.56 (0.02–1.02)	0.50 (0.16–1.50)
Sleep disturbance	0	270	1.00 (Reference)	1.00 (Reference)	119	1.00 (Reference)	1.00 (Reference)
	1	422	1.12 (0.66–1.17)	1.14 (0.59–2.21)	331	1.02 (0.24–3.56)	1.00 (0.29–3.44)
	2–3	143	1.50 (0.50–4.53)	1.46 (0.46–4.60)	100	1.82 (1.06–3.19)	1.78 (0.38–8.33)
Use of sleep medication	0	221	1.00 (Reference)	1.00 (Reference)	150	1.00 (Reference)	1.00 (Reference)
	1	428	1.16 (0.68–1.32)	1.14 (0.72–1.81)	254	0.95 (0.28–2.95)	0.97 (0.51–1.84)
	2–3	186	1.58 (0.86–4.12)	1.51 (0.82–2.79)	146	1.41 (0.69–2.80)	1.44 (0.66–3.16)
Daytime dysfunction	0	328	1.00 (Reference)	1.00 (Reference)	191	1.00 (Reference)	1.00 (Reference)
	1	372	1.24 (0.74–1.89)	1.26 (0.80–2.00)	264	1.96 (1.62–2.52)	1.94 (0.99–3.82)
	2–3	135	1.75 (0.97–3.16)	1.79 (1.00–3.19)	95	1.54 (0.55–4.97)	1.50 (0.67–3.36)

PSQI, Pittsburgh Sleep Quality Index. CI, confidence interval; **^†^** Adjusting for age, BMI, TC,TG, FBG, exercise habit, status of smoking, status of drinking, score of GAD-7 and PHQ-9.

**Table 5:** Odds ratios (OR) of sleep duration for hypertension prevalence after adjusting for the components of PSQI measuring qualitative aspects of sleep.

PSQI and Its Components	Grade of Sleep Duration	N	Unadjusted OR (95% CI)	Adjusted OR(95% CI) ^†^
Males ≤ 45 years	0	1298	1.00 (Reference)	1.00 (Reference)
	1	487	1.01 (0.75–1.36)	0.96 (0.69–1.34)
	2–3	149	1.64 (0.97–2.48)	1.60 (0.90–2.82)
Males > 45 years	0	1403	1.00 (Reference)	1.00 (Reference)
	1	536	1.05 (0.98–1.07)	1.03 (0.82–1.29)
	2–3	203	1.34 (0.91–1.95)	1.29 (0.89–1.86)

PSQI, Pittsburgh Sleep Quality Index. CI, confidence interval; **^†^** Adjusting for age, BMI, TC,TG, FBG, exercise habit, status of smoking, status of drinking, score of GAD-7, score of PHQ-9 and the total scores of six components of PSQI including subjective sleep quality, sleep latency, habitual sleep efficiency, sleep disturbance, use of sleep medication and daytime function.

## 6. Discussion

Taking both sleep quantity and quality into consideration, we used PSQI to assess the global sleep status in the present study and explored the potential association of PSQI score as well as its components with hypertension prevalence in Chinese adults. Our results demonstrated that the associations of global sleep status as well as short sleep duration with hypertension prevalence varied with age and sex. Poor sleep status was associated with hypertension prevalence in male subjects of all ages and in female subjects aged ≤45 years. Short sleep duration was found to be related to hypertension only in male subjects. However, after adjusting for the sleep qualitative aspect of PSQI on the basis of the traditional cardiovascular characteristics, the relation between short sleep duration and hypertension failed to reach a statistical significance.

One of the main findings of the present study was that sleep should be measured qualitatively and quantitatively when investigating its potential association with hypertension, and previous reports about sleep duration and hypertension may be biased by sleep quality. Dozens of studies have investigated the relationship between sleep duration and hypertension prevalence or incidence in populations of different races, genders or ages [1,2,3,4,5,22,23,24]. Most of these studies suggest a robust relationship between sleep duration and hypertension although obvious contradictions can be observed among them. To our knowledge, the relationship between sleep duration and hypertension has never been elucidated in Chinese adults before. In the present study, male and female subjects were divided into two age groups with the age of 45 years as the demarcation point. We found that short sleep duration was associated with hypertension only in men. Our results that short sleep duration was not in relation to hypertension prevalence in women were supported by a couple of previous studies conducted in the American, Japanese, Dutch, Brazilian and Spanish populations [25,26,27,28]. However, some results from other studies were contradictory to ours [3,21,23,29]. Although differences in the basic characteristics of the enrolled participants and the confounder adjusted in those studies partly explains the inconsistent associations between sleep duration and hypertension prevalence in the current literature, failing to preclude the potential confounding effects of sleep quality on hypertension may be one essential underlying reason for the discrepancy. Sleep has both quantitative and qualitative aspects. Most studies about sleep and hypertension only measured sleep duration which, in our opinion, is not comprehensive. Two previous studies which paid attention to the role of sleep quality demonstrated that examining only short sleep duration without the presentation of other forms of sleep disorders failed to exert an influence on hypertension prevalence [7,30]. In fact, short sleep duration is often accompanied by poor sleep quality. The analysis of the subjects enrolled in the present study has shown that the percentage of sleepers with poor subjective sleep quality kept increasing when sleep duration decreased from 8 h to less than 6 h (Supplementary Figure S1: the percentages of high-quality sleep in subjects with sleep duration of 8 h, 7 h, 6 h and less than 6 h in all participants). Furthermore, results from this study also indicated that poor sleep quality was also related to hypertension prevalence. These findings made the association of short sleep duration in relation to hypertension from previous studies become less reliable. To ensure the role of short sleep duration in hypertension prevalence, we believe it is necessary to adjust for the confounding effect of sleep quality. We made this adjustment in the present study and the significant association between sleep duration and hypertension before the adjustment of the qualitative aspect of sleep failed to reach a statistical significance.

Another finding of the present study was the association between global sleep status and hypertension in females: sleep status was not associated with hypertension prevalence in those aged more than 45 years. Although the potential mechanism under such a discrepancy is still unknown to us at the present time, it may result from the changes of endocrine hormones, as those aged >45 years were close to or already in the perimenopausal period and were therefore more vulnerable to major hormonal fluctuations [31,32].

OSAS and RLS could significantly increase the risk for hypertension [33,34] and they are possible confounding factors in the relationship between sleep status and hypertension prevalence. To evaluate the specific effect of sleep status on hypertension prevalence, OSAS and RLS were excluded in this study. It has been reported that snoring has a high sensitivity (87%) for detecting OSAS in previous research [35]. Thus, we excluded all subjects with suspected OSAS who snored more than one day per week, as reported by himself or roommates. In addition, RLS was excluded on the basis of self-reported (or reported by roommates) symptoms, as the diagnosis of RLS was mainly based on clinical symptoms.

Our study has several limitations. First of all, this study was designed as a cross-sectional one and did not allow the establishment of causality of the observed associations. Given the self-reporting of sleep status, we could neither preclude the possibility of the potential effect of hypertension on sleep habits and quality nor *vice versa*. Secondly, as a further limitation, because we did not have suitable questionnaires or other forms of measurement, we could not control the potential influence of eating habits on sleep disorders and hypertension, despite food being one important mediating factor in the relationship between them. Thirdly, although the sample size of the female participants was enough to yield sound and stable results, it is better to adopt a cautious attitude toward the sex differentiation for association of sleep quality and hypertension considering the high ratio of males to females in the current study.

## 7. Conclusions

The association of global sleep status evaluated by the PSQI scale and hypertension prevalence in Chinese adults varies with age and sex. In addition, the interaction of sleep duration and sleep quality needs to be addressed when investigating the association between sleep duration and hypertension. Moreover, comprehensive evaluation of sleep from qualitative and quantitative aspects will achieve better accuracy.

## Supplementary Files

Supplementary File 1

## References

[1] Bjorvatn B., Sagen I.M., Oyane N., Waage S., Fetveit A., Pallesen S., Ursin R. (2007). The association between sleep duration, body mass index and metabolic measures in the hordaland health study. J. Sleep Res..

[2] Fang J., Wheaton A.G., Keenan N.L., Greenlund K.J., Perry G.S., Croft J.B. (2012). Association of sleep duration and hypertension among US adults varies by age and sex. Amer. J. Hypertens..

[3] Cappuccio F.P., Stranges S., Kandala N.B., Miller M.A., Taggart F.M., Kumari M., Ferrie J.E., Shipley M.J., Brunner E.J., Marmot M.G. (2007). Gender-specific associations of short sleep duration with prevalent and incident hypertension: The whitehall II study. Hypertension.

[4] Gangwisch J.E., Heymsfield S.B., Boden-Albala B., Buijs R.M., Kreier F., Pickering T.G., Rundle A.G., Zammit G.K., Malaspina D. (2006). Short sleep duration as a risk factor for hypertension: Analyses of the first national health and nutrition examination survey. Hypertension.

[5] Wells J.C., Hallal P.C., Reichert F.F., Menezes A.M., Araujo C.L., Victora C.G. (2008). Sleep patterns and television viewing in relation to obesity and blood pressure: Evidence from an adolescent brazilian birth cohort. Int. J. Obes..

[6] Kaneita Y., Uchiyama M., Yoshiike N., Ohida T. (2008). Associations of usual sleep duration with serum lipid and lipoprotein levels. Sleep.

[7] Bansil P., Kuklina E.V., Merritt R.K., Yoon P.W. (2011). Associations between sleep disorders, sleep duration, quality of sleep, and hypertension: Results from the national health and nutrition examination survey, 2005 to 2008. J. Clin. Hypertens..

[8] Vargas P.A., Flores M., Robles E. (2014). Sleep quality and body mass index in college students: The role of sleep disturbances. J. Amer. Coll. Health.

[9] Logue E.E., Scott E.D., Palmieri P.A., Dudley P. (2014). Sleep duration, quality, or stability and obesity in an urban family medicine center. J. Clin. Sleep Med..

[10] Okubo N., Matsuzaka M., Takahashi I., Sawada K., Sato S., Akimoto N., Umeda T., Nakaji S. (2014). Relationship between self-reported sleep quality and metabolic syndrome in general population. BMC Public Health.

[11] Lou P., Chen P., Zhang L., Zhang P., Chang G., Zhang N., Li T., Qiao C. (2014). Interaction of sleep quality and sleep duration on impaired fasting glucose: A population-based cross-sectional survey in China. BMJ Open.

[12] Buysse D.J., Reynolds C.F., Monk T.H., Berman S.R., Kupfer D.J. (1989). The Pittsburgh sleep quality index: A new instrument for psychiatric practice and research. Psychiat. Res..

[13] Wu J., Zhang Q., Yang H., Gao X., Zhou Y., Wang A., Wang C., Zhang S., Wu S., Zhao X. (2013). Association between non-high-density-lipoprotein-cholesterol levels and the prevalence of asymptomatic intracranial arterial stenosis. PLoS One.

[14] Xue H., Wang J., Hou J., Zhu H., Gao J., Chen S., Wang Y., Chen Y., Wu S. (2013). Association of ideal cardiovascular metrics and serum high-sensitivity c-reactive protein in hypertensive population. PLoS One.

[15] Chobanian A.V., Bakris G.L., Black H.R., Cushman W.C., Green L.A., Izzo J.L., Jones D.W., Materson B.J., Oparil S., Wright J.T. (2003). Seventh report of the joint national committee on prevention, detection, evaluation, and treatment of high blood pressure. Hypertension.

[16] Tsai P.S., Wang S.Y., Wang M.Y., Su C.T., Yang T.T., Huang C.J., Fang S.C. (2005). Psychometric evaluation of the Chinese version of the Pittsburgh sleep quality index (CPSQI) in primary insomnia and control subjects. Qual. Life Res..

[17] Donker T., van Straten A., Marks I., Cuijpers P. (2011). Quick and easy self-rating of generalized anxiety disorder: Validity of the dutch web-based GAD-7, GAD-2 AND GAD-SI. Psychiat. Res..

[18] Zhang Y., Ting R., Lam M., Lam J., Nan H., Yeung R., Yang W., Ji L., Weng J., Wing Y.K. (2013). Measuring depressive symptoms using the patient health questionnaire-9 in Hong Kong Chinese subjects with type 2 diabetes. J. Affect. Disord..

[19] Han C., Jo S.A., Kwak J.H., Pae C.U., Steffens D., Jo I., Park M.H. (2008). Validation of the patient health questionnaire-9 Korean version in the elderly population: The Ansan geriatric study. Compr. Psychiat..

[20] Spitzer R.L., Kroenke K., Williams J.B., Lowe B. (2006). A brief measure for assessing generalized anxiety disorder: The GAD-7. Arch. Intern. Med..

[21] Kim J., Jo I. (2010). Age-dependent association between sleep duration and hypertension in the adult Korean population. Amer. J. Hypertens..

[22] Choi KM L.J., Park H.S., Baik S.H., Choi D.S., Kim S.M. (2008). Relationship between sleep duration and the metabolic syndrome: Korean national health and nutrition survey 2001. Int. J. Obes..

[23] Faraut B., Touchette E., Gamble H., Royant-Parola S., Safar M.E., Varsat B., Leger D. (2012). Short sleep duration and increased risk of hypertension: A primary care medicine investigation. J. Hypertens..

[24] Magee C.A., Kritharides L., Attia J., McElduff P., Banks E. (2012). Short and long sleep duration are associated with prevalent cardiovascular disease in Australian adults. J. Sleep Res..

[25] Hall M.H., Muldoon M.F., Jennings J.R., Buysse D.J., Flory J.D., Manuck S.B. (2008). Self-reported sleep duration is associated with the metabolic syndrome in midlife adults. Sleep.

[26] Knutson K.L., van Cauter E., Rathouz P.J., Yan L.L., Hulley S.B., Liu K., Lauderdale D.S. (2009). Association between sleep and blood pressure in midlife: The cardia sleep study. Arch. Intern. Med..

[27] Lima-Costa M.F., Peixoto S.V., Rocha F.L. (2008). Usual sleep duration is not associated with hypertension in Brazilian elderly: The Bambui health aging study (BHAS). Sleep Med..

[28] Van den Berg J.F., Tulen J.H., Neven A.K., Hofman A., Miedema H.M., Witteman J.C., Tiemeier H. (2007). Sleep duration and hypertension are not associated in the elderly. Hypertension.

[29] Gangwisch J.E., Feskanich D., Malaspina D., Shen S., Forman J.P. (2013). Sleep duration and risk for hypertension in women: Results from the nurses’ health study. Amer. J. Hypertens..

[30] Fernandez-Mendoza J., Vgontzas A.N., Liao D., Shaffer M.L., Vela-Bueno A., Basta M., Bixler E.O. (2012). Insomnia with objective short sleep duration and incident hypertension: The Penn State cohort. Hypertension.

[31] Parry B.L., Newton R.P. (2001). Chronobiological basis of female-specific mood disorders. Neuropsychopharmacology.

[32] Sowers M.R., la Pietra M.T. (1995). Menopause: Its epidemiology and potential association with chronic diseases. Epidemiol. Rev..

[33] Somers V.K., White D.P., Amin R., Abraham W.T., Costa F., Culebras A., Daniels S., Floras J.S., Hunt C.E., Olson L.J. (2008). Sleep apnea and cardiovascular disease: An American heart association/American college of cardiology foundation scientific statement from the American heart association council for high blood pressure research professional education committee, council on clinical cardiology, stroke council, and council on cardiovascular nursing. In collaboration with the national heart, lung, and blood institute national center on sleep disorders research (national institutes of health). Circulation.

[34] Batool-Anwar S., Malhotra A., Forman J., Winkelman J., Li Y., Gao X. (2011). Restless legs syndrome and hypertension in middle-aged women. Hypertension.

[35] Pedrosa R.P., Drager L.F., Gonzaga C.C., Sousa M.G., de Paula L.K., Amaro A.C., Amodeo C., Bortolotto L.A., Krieger E.M., Bradley T.D., Lorenzi-Filho G. (2011). Obstructive sleep apnea: The most common secondary cause of hypertension associated with resistant hypertension. Hypertension.

