# Supplementary material for: Association between Self-Reported Global Sleep Status and Prevalence of Hypertension in Chinese Adults: Data from the Kailuan Community
Source: Int J Environ Res Public Health. 2015 Jan 7;12(1):488–503. doi: 10.3390/ijerph120100488 (PMC4306875; doi:10.3390/ijerph120100488)
Supplement: Supplementary File 1 [file ijerph-12-00488-s001.pdf]

Supplementary Information

Association between Self-Reported Global Sleep Status and Prevalence of Hypertension in Chinese Adults: Data from the Kailuan Community

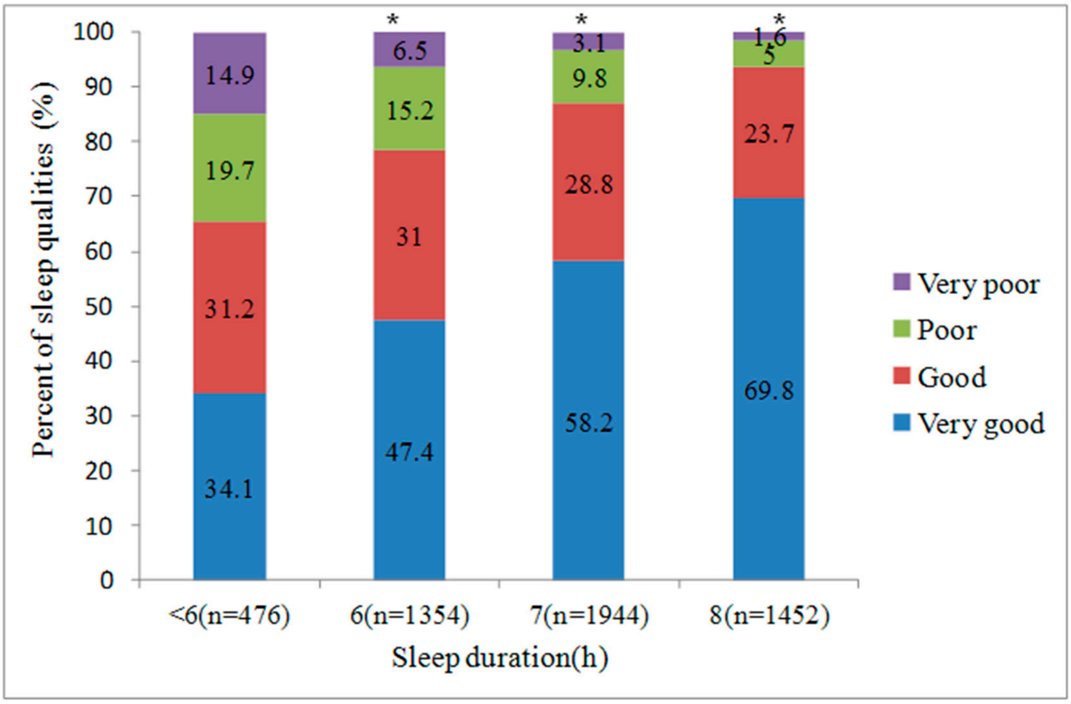

Figure S1. The percentages of sleep quality in subjects with sleep duration of 8 h, 7 h, 6 h and less than 6 h in all participants.

© 2015 by the authors; licensee MDPI, Basel, Switzerland. This article is an open access article distributed under the terms and conditions of the Creative Commons Attribution license (http://creativecommons.org/licenses/by/4.0/).
